# Authorship disintegrity in research collaborations: ends do not justify means in science

**DOI:** 10.3402/meo.v19.24930

**Published:** 2014-06-17

**Authors:** Ahmed Abu-Zaid, Asma Alnajjar, Lucman A. Anwer

**Affiliations:** College of Medicine, Alfaisal University, Riyadh, Saudi Arabia

In today’s competitive academic climate, academic medical educators are under constant pressure to generate scholarship for promotion and job security. Similarly, for undergraduate medical students, research publications are viewed as valued assets for gaining acceptance into quality postgraduate medical programs. Not surprisingly, one of the most pragmatic routes to glean this highly desired currency is through research collaborations as measured by coauthored publications (1).

Aside from the aforementioned benefits, such collaborations also offer: 1) valuable experience in modern, often complex research; 2) optimal use of related, sometimes scarce resources; 3) effective transmission of research knowledge and skills; 4) potential dissemination of published work; 5) opportunities to cross-pollinate novel research ideas/insights; 6) networking and team building; and 7) a general ‘socialization’ into the research arena.

With the growth of research collaborations, authorship (dis)integrity in such partnerships remains a key topic of discussion. For example, the International Committee of Medical Journal Editors (ICMJE) provides guidelines for distinguishing authors (liable for all aspects of the research process and to whom credit should be given) from nonauthors (2). Previously, the ICMJE required that authorship be based on all of the following criteria: 1) substantial contributions to the conception or design of the work – or the acquisition, analysis, or interpretation of data for the work; 2) drafting or critically revising the work for important intellectual content; and 3) final approval of the version to be published. A more recent addition, ‘Agreement to be accountable for all aspects of the work in ensuring that questions related to the accuracy or integrity of any part of the work are appropriately investigated and resolved’, appears intended to deter unethical authorship practices (2).

However, discouraging undue credit on author bylines is difficult, and current practices reflect the pressures on scholars to be ‘productive’. One example of ‘unjustified authorships’ includes listing ‘noncontributing collaborators’ for social or personal gain (e.g., assuming a favor granted now will be reciprocated in a future publication). Another such strategy involves including renowned or well-established scholars as ‘honorary (guest) authors’ to facilitate dissemination in respected outlets. Finally, and arguably more common, is the granting of shared authorship – rather than simply acknowledging – to ‘minimally contributing research collaborators’.

The possibility of a rising trend in authorship (dis)integrity among research collaborators begs the question: Who is ultimately responsible for maintaining authorship integrity in such instances? While professional or organizational oversight bodies do play important roles in monitoring and enforcing an ethical standard, these should be considered secondary roles; ideally, the primary responsibility comes down to the research collaborators themselves – with ethical behaviors originating at the individual level.

Certainly, input by external regulatory bodies, journal editors, medical educators, and researchers are needed to effectively explore and successfully cultivate authorship integrity in research collaborations involving all levels of participants – from medical students to senior researchers. Indeed, integrating into undergraduate medical curricula formal ‘codes of ethics in research collaboration’ is a good first step in fostering the notion of authorship integrity in research collaborations.

Authorship (dis)integrity remains a form of scientific fraud that should not be dealt with lightly. Realistically, however, until opposing cultural and economic pressures equalize, relying upon collaborators to assign shared credit justly and ethically will be an imperfect safeguard to ensuring that, in science, ‘the ends do not justify the means’.
